# Classification of Hematoxylin and Eosin-Stained Breast Cancer Histology Microscopy Images Using Transfer Learning with EfficientNets

**DOI:** 10.1155/2021/5580914

**Published:** 2021-04-09

**Authors:** Chanaleä Munien, Serestina Viriri

**Affiliations:** School of Mathematics, Statistics and Computer Science, University of KwaZulu-Natal, Durban 217013433, South Africa

## Abstract

Breast cancer is a fatal disease and is a leading cause of death in women worldwide. The process of diagnosis based on biopsy tissue is nontrivial, time-consuming, and prone to human error, and there may be conflict about the final diagnosis due to interobserver variability. Computer-aided diagnosis systems have been designed and implemented to combat these issues. These systems contribute significantly to increasing the efficiency and accuracy and reducing the cost of diagnosis. Moreover, these systems must perform better so that their determined diagnosis can be more reliable. This research investigates the application of the EfficientNet architecture for the classification of hematoxylin and eosin-stained breast cancer histology images provided by the ICIAR2018 dataset. Specifically, seven EfficientNets were fine-tuned and evaluated on their ability to classify images into four classes: *normal, benign, in situ carcinoma,* and *invasive carcinoma*. Moreover, two standard stain normalization techniques, Reinhard and Macenko, were observed to measure the impact of stain normalization on performance. The outcome of this approach reveals that the EfficientNet-B2 model yielded an accuracy and sensitivity of 98.33% using Reinhard stain normalization method on the training images and an accuracy and sensitivity of 96.67% using the Macenko stain normalization method. These satisfactory results indicate that transferring generic features from natural images to medical images through fine-tuning on EfficientNets can achieve satisfactory results.

## 1. Introduction and Background

One of the leading causes of death in women throughout the world is breast cancer [1]. It is defined as a group of diseases in which cells within the tissue of the breast alter and divide in an uncontrolled manner, generally resulting in lumps or growths. This type of cancer often begins in the milk glands or ducts connecting these glands to the nipple. In the beginning stages of the illness, the small tumour that appears is much easier to treat effectively, averting the disease's progression and decreasing the morbidity rates; this is why screening is crucial for early detection [2].

The process of breast cancer diagnosis begins with palpation, periodic mammography, and ultrasonic imaging inspection. The results of these procedures indicate whether further testing is required. If cancer is suspected in a patient, a biopsy is performed and tissue for microscopic analysis is procured so that a pathologist may conduct a histological examination of the extracted tissue to confirm the diagnosis [2, 3]. Once the biopsy is complete, the tissue is analyzed in a laboratory. The tissue preparation process must begin with formalin fixation and, after that, embedding in paraffin sections. The paraffin blocks are then sliced and fixed on glass slides. Unfortunately, interesting structures such as the cytoplasm and nuclei in the tissue are not yet apparent at this point. The lack of clarity in the tissue necessitates staining of the tissue so that the structures can become more visible. Typically, a standard and well-known staining protocol, using hematoxylin and eosin, is applied. When added to the tissue, the hematoxylin can bind itself to deoxyribonucleic acid, which results in the nuclei in the tissue being dyed a blue/purple color. On the other hand, the eosin can bind itself to proteins, and, as a result, other relevant structures such as the stroma and cytoplasm are dyed a pink color. Traditionally, after staining, the glass slide is coverslipped and forwarded to a pathologist for examination [4]. Routinely, the expert gathers information on the texture, size, shape, organization, interactions, and spatial arrangements of the nuclei. Additionally, the variability within, density of, and overall structure of the tissue is analyzed. In particular, the information concerning the nuclei features is relevant for distinguishing between noncarcinoma and carcinoma cells. In contrast, the information concerning the tissue structure is relevant for distinguishing between *in situ* and invasive carcinoma cells [5].

**Figure 1:**
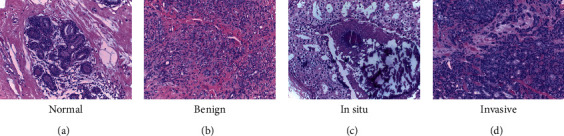
Different classes of histology microscopy images: (a) normal; (b) benign; (c) in situ; (d) invasive.

The noncarcinoma class consists of normal tissue and benign lesions; these tissues are nonmalignant and do not require immediate medical attention. *In situ* and invasive carcinoma, on the other hand, are malignant and become continuously more lethal without treatment. Specifically, *in situ* carcinoma refers to the presence of atypical cells that are confined to the layer of tissue in the breast from which it stemmed. Invasive carcinoma refers to the presence of atypical cells that invades the surrounding normal tissue, beyond the glands or ducts from where the cells originated [2]. Invasive carcinoma is complicated to treat, as it poses a risk to the entire body [3]. This threat means that the odds of surviving this level of cancer decreases as the progression stages increase. Moreover, without proper and adequate treatment, a patient's *in situ* carcinoma tissue can develop into invasive carcinoma tissue. Therefore, it is of paramount importance that biopsy tissue is examined correctly and efficiently so that a diagnosis can be confirmed and, subsequently, treatment can begin. Examples of histology images belonging to each of these classes are shown in Figure 1.

The task of performing a practical examination on the tissue is not simple and straightforward. On the contrary, it is rather time-consuming and, above all, prone to human error. The average diagnostic accuracy between professionals is around 75% [6]. These issues can result in severe and fatal consequences for patients who are incorrectly diagnosed [7].

The advancement of image acquisition devices that create whole slide images (WSI) from scanning conventional glass slides has promoted digital pathology [8]. The field of digital pathology focuses on bringing improvement in accuracy and efficiency to the pathology practice [9] by associating histopathological analysis with the study of WSI [8].

An excellent solution to address the limitations of human diagnosis is computer-aided diagnosis (CAD) systems, which are developed to automatically analyze the WSI and provide a potential diagnosis based on the image. These systems currently contribute to improving efficiency and reducing both the cost of diagnosis and interobserver variability [5, 10]. Even though current CAD systems that operate at high sensitivity provide relatively good performance, they will remain a second-opinion clinical procedure until the performance is significantly improved [10].

Recently, deep learning approaches to the development of CAD systems have produced promising results. Previous attempts to classify breast cancer histology images using a combination of handcrafted feature extraction methods and traditional machine learning algorithms required additional knowledge and were time-consuming to develop. Conversely, deep learning methods automate this process. These systems allow pathologists to focus on difficult diagnosis cases [11].

Hence, to ensure early diagnosis in breast cancer candidates, increase treatment success, and lower mortality rates, early detection is imperative. Although the advent of and advancements in computer-aided systems have benefited the medical field, there is plenty of room for improvement.

### 1.1. Research Problem

In general, the shortage of available medical experts [12], the time-consuming quest to reach a final decision on a diagnosis, and the issue of interobserver variability justify the need for a system that can automatically and accurately classify breast cancer histopathology images. Previous approaches to this problem have been relatively successful considering the available data and return adequate classification accuracies but tend to be computationally expensive. Thus, this work will explore the use of seven lightweight architectures within the EfficientNet family [13]. Since the EfficientNet models were designed to optimize available resources, while maintaining high accuracies, a CAD system that performs at the level of the current state-of-the-art deep learning approaches, while consuming less space and training time, is desirable. Transfer learning techniques have become a popular addition to deep learning solutions for classification tasks. In particular, many state-of-the-art approaches utilize fine-tuning to enhance performance [14]. Therefore, this research explores the application of seven pretrained EfficientNets for the classification of breast cancer histology images. Furthermore, the addition of stain normalization to the preprocessing step will be evaluated. Hence, the primary question that this research will answer is, “Can fine-tuned EfficientNets achieve similar results to current state-of-the-art approaches for the application of classifying breast cancer histology images?”

### 1.2. Research Contributions

In this research, the application of seven versions of EfficientNets with transfer learning for breast cancer histology image classification is investigated. The proposed architecture was able to effectively extract and learn the global features in an image, such as the tissue and nuclei organization. Of the seven models tested, the EfficientNet-B2 architecture produced superior results with an accuracy of 98.33% and sensitivity of 98.44%.

The key takeaway from this investigation is that the simple and straightforward approach to using EfficientNets for the classification of breast cancer histology images reduces training time while maintaining similar accuracies to previously proposed computationally expensive approaches.

### 1.3. Paper Structure

The remainder of the paper is structured as follows: Section 2, the literature review, provides details on previous successful approaches.  Section 3, the methods and techniques, provides insight into the framework followed in this study.  Section 4, the results, provides details of the results that were obtained during the research. Finally, section 5 elaborates on the insights of this work and concludes the paper.

## 2. Literature Review

Currently, computer-aided diagnosis (CAD) systems occupy the position of aiding physicians during the process of diagnosis, by easing their workload and reducing the disagreement that stems from the subjective interpretation of pathologists. However, the performance of these systems must be enhanced before they can be considered more dependable than a second-opinion system [10].

### 2.1. Traditional Approaches

In the traditional approach, expert domain knowledge is required so that the correct features may be handcrafted; this is a time-consuming endeavor. Nevertheless, the approach yields acceptable results on the datasets used. For instance, Kowal [15] used multiple clustering algorithms to achieve nuclei segmentation on microscopic images. Segmentation made it possible to extract microscopic, textural, and topological features so that classifiers could be trained and images could be classified as either benign or malignant. The accuracy of patient-wise classification was in the range of 96–100%. It is worth noting that this method performs poorly when an image contains overlapping nuclei or a small number of nuclei. In this case, either the approach fails to identify the nuclei or the clustering algorithms could return unreliable results. Therefore, in order to attain an acceptable detection accuracy, a large number of sample images are required. Hence, it is evident that accurate nuclei segmentation is not a straightforward task; this can also be attributed to the variability in tissue appearance or the presence of clustered or tightly clumped nuclei [5].

An alternative approach is utilizing information on tissue organization as in the work by Belsare et al. [16], which presents a framework to classify images into malignant and nonmalignant. Firstly, segmentation was done using spatio-color-texture graphs. After that, statistical feature analysis was employed, and classification was achieved with a linear discriminant classifier. The choice of this classifier considerably impacted the outcome of this approach, as the result outperformed the use of k-nearest-neighbor and state vector machine classifiers, especially for the detection of nonmalignant tissue. Accuracies of 100% and 80% were achieved for nonmalignant and malignant images, respectively.

### 2.2. Deep Learning Approaches

The increase in the availability of computing power has led to the emergence of advanced architectures called convolutional neural networks (CNNs). Contrary to the conventional approach, no expert domain knowledge is required to define algorithms for segmentation, feature extraction, and classification, but instead expert knowledge is needed to annotate the dataset for a CNN to achieve superior results. Instead, these networks can automatically determine and extract discriminative features in an image that contribute to the classification of the image. Generally, a CNN will use a training set of images to learn features that are unique to each class so that when a similar feature is detected in an unseen image, the network will be able to assign the image to a class with confidence.

#### 2.2.1. Convolutional Neural Network Approaches

The success of convolutional neural networks (CNNs) with general computer vision tasks motivated researchers to employ these models for classifying histopathology images. For the classification of hematoxylin and eosin-stained breast cancer histology images, both Araújo et al. [5] and Vo et al. [17] used the Bioimaging 2015 dataset [18] and classified the images into four classes (normal, benign, *in situ*, and invasive) and two groups (carcinoma and noncarcinoma). The former work [5] proposes a CNN that can integrate information from multiple histological scales. The process begins with stain normalization via the method proposed by Macenko et al. [19] in a bid to correct color discrepancies. After that, 12 512 × 512 overlapping patches were extracted from each image. The chosen size of the patches ensures that no relevant information is lost during extraction and, therefore, every patch can be appropriately labeled. Then, data augmentation was used to increase the number of images in the dataset. Finally, a patch-wise trained CNN and a fusion of a CNN and support vector machine classifier (CNN + SVM) were used to determine the patch class probability. Image-wise classification was attained through a patch probability fusion method. The evaluation showed that using majority voting strategy as the fusion method produced the best results. Considering all four classes, patch-wise classification with the CNN achieved an accuracy rate of 66.7%, while the CNN + SVM achieved an accuracy rate of 65%. The image-wise classification achieved higher results at 77.8% accuracy for both classifiers. With only two classes, patch-wise accuracy for the CNN was 77.6%, and for the CNN + SVM, the approach yielded 76.9% accuracy. The image-wise classification for the 2-class task produced the best results at 80.6% for the CNN and 83.3% for the CNN + SVM. The reason for the lower patch-wise classification is that images may contain sections of normal-looking tissue. Since during patch generation the extracted patches inherit the image's label, this may confuse the CNN. The increase in image-wise classification accuracy is due to the fusion method that is applied. The authors also recorded the sensitivity rates for each of the classes. It is worth noting that overall, for image-wise classification, the approach was more sensitive to the carcinoma class than the noncarcinoma class. This outcome, although not ideal, is preferable since the architecture that was proposed focuses on correctly classifying the carcinoma (malignant) instances [5].

The approach taken by Vo and Nguyen [17] proposed a combination of an ensemble of deep CNNs and gradient boosting tree classifiers (GBTCs). Stain normalization via Macenko et al. [19] and data augmentation were the initial steps of the process. Unlike the standard data augmentation method of rotating and flipping images, the proposed method [17] incorporates reflection, translation, and random cropping of the images. The normalized and augmented data was then used to train the proposed architecture. Specifically, three deep CNNs (Inception-ResNet-v2) were trained using three different input sizes: 600 × 600,450 × 450, and 300 × 300. Then, visual features were extracted and fed into GBTCs, which increased classification performance. The majority voting strategy was used to merge the outputs of the GBTCs, resulting in a much more robust solution. Recognition rates of 96.4% for the 4-class classification and 99.5% for the 2-class classification were reported. This result surpasses state-of-the-art achievements. An interesting note is that the authors added global average pooling layers in place of dense (fully connected) layers, and this did not negatively impact the accuracy of the ensemble. Similar to Araújo et al. [5], the authors of this work recorded the sensitivities of their approach. The results indicate that, for the 4-class task, the proposed method struggles with the classification of the *in situ* instances, while the other three classes have incredibly high sensitivities. For the 2-class task, the approach yields a 100% sensitivity on carcinoma instances and 98.9% on noncarcinoma instances. These results indicate that the approach was able to successfully learn both local and global features for the multiclass and binary classification. However, the downfall of this approach is the computational expense.

#### 2.2.2. Convolutional Neural Network with Transfer Learning Approaches

For the TK-AlexNet proposed by Nawaz et al. [3] to classify breast cancer histology images, the classification layers of the AlexNet architecture were replaced with a single convolutional layer, and a max-pooling layer was added before the three fully connected layers with 256, 100, and 4 neurons, respectively. The input size of the proposed network [3] was increased to 512×512. The transfer learning technique used in this application was to fine-tune the last three layers on the ICIAR2018 dataset after having the entire network trained on the ImageNet dataset. The images were stain-normalized with the method proposed in Macenko et al. [19]. An interesting fact is that the authors compared the performance of the model with both non-stain-normalized and stain-normalized images and concluded that using the latter resulted in a gain in performance. After that, data augmentation techniques such as mirroring and rotation were applied, and overlapping patches of size 512 × 512 were extracted from each image. Hence, there was a total of 38400 images generated. Evaluation of the model was done using a train-test split of 80%–20%.

The image-wise accuracy reported in [3] was 81.25%, and the patch-wise accuracy was 75.73%. A noteworthy observation is that the normal and benign classes were classified with 85% sensitivity; however, the *in situ* and invasive carcinoma classes were classified with 75% sensitivity. For a model to be practical as a second-opinion system, it should ideally have a higher sensitivity to the carcinoma class given the dangers of misdiagnosis.

For this classification task, the Inception-ResNet-v2 was used by Ferreria et al. [20]. The classification layers of the base model were replaced by a global average pooling layer, a dense (or fully connected) layer with 256 neurons, a dropout layer with a dropout rate of 0.5, and a final dense layer of 4 neurons. Moreover, the input size of the network was changed to 244 × 244. Reshaping the images does not significantly impact the form of the cellular structures; however, it does reduce computational cost [20]. The authors did not incorporate stain normalization into their experiments. Data augmentation techniques such as image flips (horizontal and vertical), a 10% zoom range, and shifts (horizontal and vertical) were used to increase the dataset. These particular techniques were chosen with care because if the augmentation causes too much distortion, the anatomical structures in the image could be destroyed [20], and this may result in the network having difficulty extracting discriminative features during training.

Two forms of transfer learning were used in this experiment. At first, only the dense (fully connected) layers of the model were trained. This technique is referred to as feature extraction since the network is using pretrained features (from ImageNet) to classify the breast cancer histology images. The result of this step is that only the weights of the dense layers were adjusted. This aids in overfitting [20]. Afterwards, a certain number of layers were unfrozen so that the network could be fine-tuned. Early stopping with a patience of 20 epochs, and a checkpoint callback monitoring minimum validation loss were the additional techniques implemented to avoid overfitting. The dataset was randomly split into 70% training, 20% validation, and 10% testing. The test set achieved an accuracy of 90%.

In a study by Kassani et al. [21], five different architectures (Inception-v3, Inception-ResNet-v2, Xception, VGG16, and VGG19) were investigated for the classification of the ICIAR2018 dataset. Two stain normalization methods were observed in this study: Macenko et al. [19] and Reinhard et al. [22]. Data augmentation included vertical flips, contrast adjustment, rotation, and brightness correction. The data was split into 75% and 25% for training and testing, respectively. The images were resized to 512 × 512 pixels with the help of bicubic interpolation. For each of the models, features were extracted from specific blocks, particularly the layer after a max-pooling layer. The extracted features were put through a global average pooling layer and then concatenated to form a feature vector which was fed into an MLP (multilayer perceptron) set with 256 neurons for final classification. Of these models, the modified Xception network trained with Reinhard stain-normalized images performed the best, with a reported accuracy score of 94%. Overall, the Xception architecture performed the best for both of the stain normalization methods, and the Reinhard [22] technique produced higher accuracies than Macenko [19]. The other architectures ranked in the following order: Inception-v3, Inception-ResNet-v2, VGG16, and VGG19. Interestingly, the approximate parameters for these architectures are 23 million, 54 million, 138 million, and 143 million, respectively. One could hypothesize that an increase in parameter count translates to a decrease in accuracy of this dataset. This indicates that the bigger architectures may have more difficulty extracting critical features from training images, even if measures are taken to enlarge the dataset being used. The results of this study also emphasize the benefit of incorporating stain normalization into preprocessing and how choosing the correct method improves accuracy significantly.  Table 1 shows a comparison summary of related deep learning techniques in the literature.

## 3. Methods and Techniques

**Figure 2:**
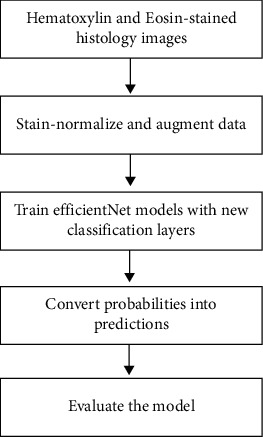
Training process of the experiments.

**Figure 3:**
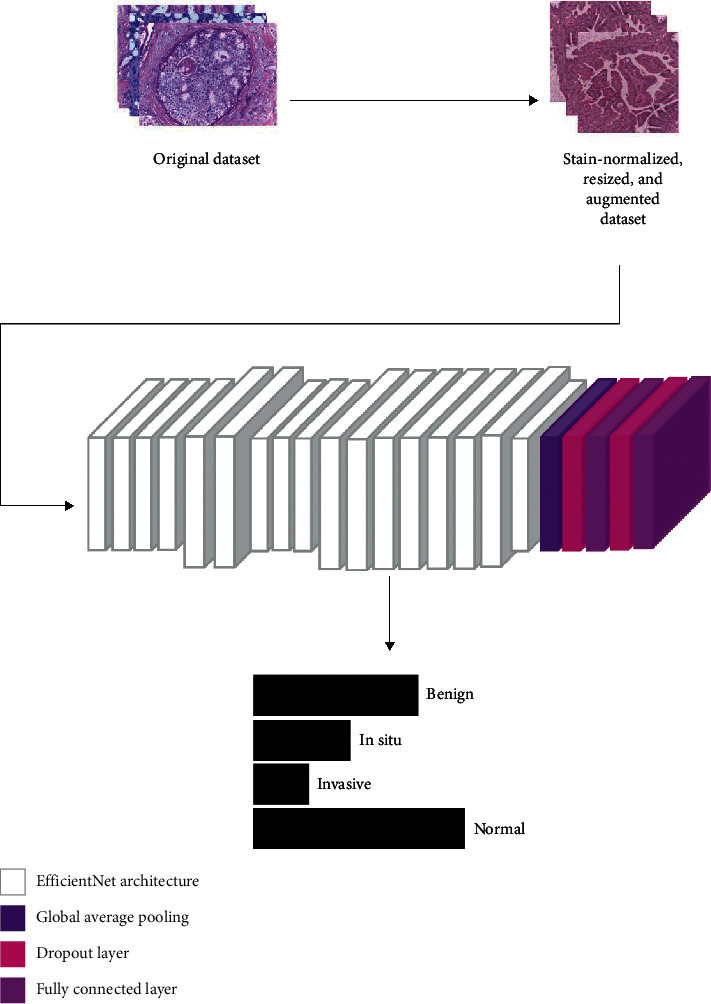
Proposed model for classification of histology microscopy images using deep learning.

The process followed in this research is depicted in Figure 2. The method consisted of two major phases. In the first phase, all the images in the ICIAR2018 dataset were stain-normalized using two techniques: Reinhard [22] and Macenko [19]. For a better understanding of the impact of stain normalization, experiments with nonnormalized images were also conducted. Then specific data augmentation techniques were randomly applied to the images. These augmentation techniques were chosen so that the images would not be too distorted, to avoid the risk of losing distinguishing features. For the second phase, the EfficientNet models were extended to perform classification. For this architecture, sufficient regularization was necessary as the dataset used was relatively small compared to what deep learning models require. This limitation introduces the possibility of overfitting, and employing regularization techniques counteracts this.  Figure 3 depicts the proposed method.

### 3.1. Dataset

The dataset used in this research is called the ICIAR2018 breast cancer histology images dataset [14] and is an extension of the 2015 Bioimaging breast cancer histology images dataset [5]. It contains 400 high-resolution microscopy images and is separated into four classes: normal, benign, *in situ* carcinoma, and invasive carcinoma. All four classes are equally represented. Two medical professionals annotated each image, and if the professionals disagreed on a particular image's annotation, the image was either discarded or confirmed through immunohistochemical analysis. The dataset is available in RGB.tiff format, and each image is 2048 × 1536 pixels in size, with a pixel scale of 0.42*μ*m × 0.42*μ*m (which refers to the area of tissue covered by a pixel) with a magnification of 200×.

### 3.2. Preprocessing

Preprocessing is crucial for the classification of histology images. The images in the dataset [14] are rather large while convolutional neural networks are typically designed to take in much smaller inputs. Therefore, the resolution of the images must be decreased so that the network is able to receive the input while maintaining the important features. The size of the dataset is much smaller than what is generally required to train a deep learning model properly; data augmentation is utilized to increase the amount of unique data in the set. This technique contributes toward avoiding overfitting, a phenomenon whereby the model learns the training data well but is entirely unable to generalize and classify unseen images.

#### 3.2.1. Stain Normalization

Many factors contribute to color inconsistencies in histology images, but they are primarily due to the tissue preparation and histology staining process. Other factors may include the conditions and small differences in the labs where the slides are prepared. The techniques used in the process and fixation delays as well as the conditions during slide digitization using a scanner, such as changes in light sources, detectors, or optics, contribute to the discrepancies [4]. These discrepancies in colors in the images could negatively impact the training process in CNNS. [23]. There have been many stain normalization techniques proposed. In this research, two techniques were applied, proposed by Reinhard et al. [22] and Macenko et al. [19].

These techniques aid in improving the efficiency and accuracy of a network by reducing the color inconsistencies in the images. Moreover, without stain normalization, the network may learn staining patterns instead of extracting the relevant features [14].

However, the majority of the top performing methods reported in the “ICIAR2018 Grand Challenge” paper [14] did not use any form of stain normalization, so we also conducted experiments with images that were not normalized.

Images must be converted from the BGR color space to the RGB color space in order for the stain normalization techniques to function as expected.

*(1) Macenko Stain Normalization.* This technique [19] accounts for the staining protocol used during the preparation of the tissue slide. Firstly, the colors are converted to optical density (OD) via the simple logarithmic transformation.

A value, *β*, is specified and used as a threshold to remove data with higher OD intensity. Singular value decomposition (SVD) is applied to the optical density tuples from the first step in order to determine a plane. This plane corresponds to the two largest singular values found. The optical density-transformed pixels are then projected onto this plane so that the angle at every point concerning the first SVD direction can be determined. Then, the color space transform resulting from the previous steps is applied to the original breast cancer histology image, and the histogram of the image is stretched such that the range covers the lower (100–*α*)% of the data. Minimum and maximum vectors are calculated and projected back into the optical density space. The hematoxylin stain corresponds to the former vector, and the eosin stain corresponds to the latter vector. The concentrations of the stains are appropriately determined, and the resulting matrix represents the RGB channels and OD intensity. The values *α* and *β* are recommended to be set to 1 and 0.15, respectively, and are kept the same for these experiments.

*(2) Reinhard Stain Normalization.* This technique [22] focuses on mapping the color distribution of an over- or under-stained image to a well-stained image. The use of linear transformation from RGB to *lαβ* color space by matching mean and standard deviation values of the color channels achieves this. Essentially, the mean color within the selected target image is transferred onto the source image. This method preserves the intensity variation of the original image. This, in turn, preserves its structure, while its contrast is adjusted to that of the target. In the *lαβ* color space, the stains are not precisely separated. The *lαβ* color space must be converted back into RGB to attain the normalized image.

**Figure 4:**
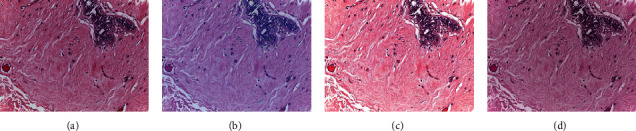
Examples of original and normalized images: (a) target image; (b) original image; (c) Macenko-normalized; (d) Reinhard-normalized.

Figure 4 shows examples of the stain normalization techniques applied in this study. In this figure, (a) represents the target image that was used for both techniques. Essentially, the techniques aim to normalize the colors in the original images to those of the target. An example of the original image is shown in (b). The subfigures (c) and (d) show the result of using (a) on (b) with the Macenko and Reinhard techniques, respectively.

#### 3.2.2. Data Augmentation

**Figure 5:**
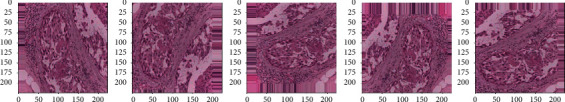
Random data augmentation applied to a normalized image.

For this step, combinations of methods that are provided by the Keras library were tested to observe the impact on overfitting and contribution to improving classification accuracy. The process of analyzing histology images is rotationally invariant, which means that, irrespective of the angle at which a pathologist views a microscopy image, he/she is still able to examine the image. Therefore, applying a rotation augmentation to the image should not negatively impact the training of the architecture. The rotation augmentation is customized (as in the approach in [5]) such that an image is rotated (90*k*)° in a clockwise direction where *k*={0,1,2,3}. Additionally, a width and height shift, zoom range, and horizontal and vertical flips were randomly applied to rescaled images. This step is implemented in a way that allows the augmentation to be done dynamically; therefore, no extra storage is required. The normalized images are randomly augmented as they are fed into the model for training.  Figure 5 shows an image normalized with Reinhard [22], resized, and randomly augmented with the methods mentioned in Table 2. The images were resized according to the recommended input size of each EfficientNet architecture as shown in Table 3. Table 3 contains the relevant information for each EfficientNet resolution. Resizing the images directly causes a loss of local features but preserves global features in the image. Therefore, the success of the experiments depend on the architecture's ability to recognize and learn these global features [23].

### 3.3. Transfer Learning

Transfer learning (TL) can be described as follows: “given a source domain *𝒟*_*𝒮*_ and learning task *𝒯*_*𝒮*_, a target domain *𝒟*_*𝒯*_ and learning task *𝒯*_*𝒯*_, transfer learning aims to help improve the learning of the target predictive function *f*_*T*_ (*·*) in *𝒟*_*𝒯*_ using the knowledge in *𝒟*_*𝒮*_ and *𝒯*_*𝒮*_, where *𝒟*_*𝒮*_  ≠  *𝒟*_*𝒯*_, or *𝒯*_*𝒮*_  ≠  *𝒯*_*𝒯*_” [24].

The earlier and middle layers of a CNN detect edges and generic shapes, while the layers toward the end of a CNN detect problem-specific features. The concept of transfer learning is based on utilizing the general features learned in the earlier layers from the source dataset, and a specified number of layers at the end of the model are retrained on the target dataset. The main benefits of TL are saving training time, improving performance of the neural network, and circumventing the limitations caused by lack of data [25]. This technique has been effective in overcoming the issue of small datasets [26].

In the work of Shallu et al. [27], the application of transfer learning for breast cancer histology image classification was investigated. The pretrained networks used in this study were VGG16, VGG19, and ResNet-50. In order to evaluate the effect of using pretrained weights with these models, the authors used the networks as feature generators, extracted features from the images, and used these features to train a logistic regression classifier. The results of these tests were then compared to the results of a full-trained network (trained from scratch with randomly initialized weights). It was proven that fine-tuning significantly impacted the reported precision, recall, F1, accuracy, AUC, and APS scores. The VGG16 model, which is the smallest model investigated (depth-wise, having only 16 convolutional layers) in the experiment, performed the best on the fine-tuning tests, reaching an accuracy of 92.6%. A supposition one can make from the reported fine-tuning results of this study is that the CNN architectures that were larger (depth-wise) had lower accuracy scores: VGG16 obtained 92.6% accuracy, VGG19 obtained 90% accuracy, and ResNet-50 obtained 79.4% accuracy with a train-test split of 90%–10%. This indicates that network capacity is an important factor to consider when choosing a network to fine-tune. A conclusion made in this study was that the fine-tuned networks were more robust to the different sizes of train-test splits than the fully trained networks were.

There are, of course, various challenges with the application of transfer learning to medical image classification, as reported in [28]. One challenge is that medical image classification tasks do not have a sufficient amount of annotated data that is available for training CNNs [29]. This can be attributed to the expense and complexity of the process of annotating images [14]. This lack of data means that large CNNs that generally perform well in applications such as ImageNet would have difficulty avoiding overfitting on these datasets. Therefore, an ample amount of regularization in different forms is needed. Overparameterization is another one of these challenges, and it refers to the great number of parameters in a network. The more trainable parameters a network has, the longer the network will require training, the larger the number of required epochs will be, and the more computation it will require. This is not ideal in the real-world application of these models. A possible way to circumvent these challenges is to use lightweight architectures that are smaller in size and have fewer parameters, which results in more efficient use of computational power [28]. EfficientNet [13], SqueezeNet [30], and MobileNet-v2 [31] are a few of the recently proposed lightweight architectures.

Multiple forms of transfer learning have been proposed. This includes weight initialization, feature extraction, and fine-tuning. For this application, empirical observations revealed that the combination of feature extraction and fine-tuning did not enhance accuracy. On the contrary, the feature extraction phase could not effectively transfer features from the source dataset to classify the breast cancer histology images. This outcome can be attributed to the fact that the source dataset consists of natural images which bear no resemblance to the histology images. Therefore, high-level features found in the pretrained model's upper layers do not contribute to this specific classification task. These experiments resulted in extreme overfitting even though the various preventative measures were taken. Hence, we can conclude that fine-tuning the architecture with the dataset and utilizing the source dataset's low-level features yield far more acceptable results in this study.

**Figure 6:**
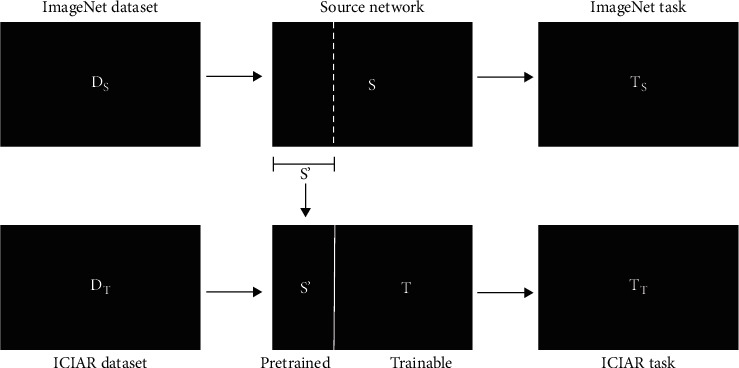
Visualization of fine-tuning.

Fine-tuning is described as freezing a certain number of layers in the model such that the generic features extracted at the beginning layers are well utilized. For this study, these generic features come from training on the ImageNet dataset [3, 20]. This dataset contains approximately 14 million natural images with 22 thousand visual categories.  Figure 6 depicts the process of fine-tuning.

Choosing the most suitable layer to begin fine-tuning from requires extensive testing. Studies such as [12] have investigated which block to tune from such that results are optimal. The authors [12] concluded that fine-tuning the top layers of a network is much more beneficial than the entire network. However, for this study, fine-tuning began at the third block. We note that beginning fine-tuning from higher blocks results in slight overfitting.

In addition to ImageNet weights, noisy-student weights [32] were also employed to train the models. Empirical observations showed that ImageNet weights were more appropriate for this application.

### 3.4. EfficientNet

The process of scaling convolutional neural networks is not well understood and is sometimes done arbitrarily until a satisfactory result is found. This process can be tedious because manual tweaking of the relevant parameters is required [13]. The earlier proposed methods of scaling a network include scaling a model by depth [33], by width [34], and by image resolution [35]. Tan and Quoc [13] studied the influence of these scaling methods in a bid to develop a more systematic way of scaling network architecture. The key findings of their research can be summarized into two specific notes: Firstly, scaling up any single dimension of network resolution, depth, or width will improve accuracy; however, this accuracy gain will diminish for larger models. Secondly, to achieve improved accuracy and efficiency, it is essential to balance the dimensions of a network's depth, width, and resolution, instead of focusing on just one of these. Considering these findings, the authors presented a novel scaling method that uses a robust compound coefficient, *ϕ*, to scale up the networks in a much more structured manner. Equation (1) represents how the authors [13] suggest scaling the depth, width, and resolution with respect to *ϕ*.

(1) $$\begin{array}{c} d=\alpha^{\phi },\\ w=\beta^{\phi },\\ r=\gamma^{\phi },\\ s.t.\;\alpha \cdot \beta^{2}\cdot \gamma^{2}\approx 2,\\ \alpha \geq 1,\beta \geq 1,\gamma \geq 1, \end{array}$$

where *d*, *w*, and *r* represent the depth, width, and resolution of the network, respectively, while the constant terms *α*,  *β*, and *γ* are determined by a hyperparameter tuning technique called grid search. The coefficient *ϕ* is user-specified and manages the resources that are available for model scaling. The constants define how the additional resources are assigned to the dimensions in the network. The “floating point operations per second” (FLOPS) is a measure of computer performance [36] and essentially measures how many operations are required to execute the network. If the network's depth is doubled, the number of FLOPS required is doubled too. If the network's width or resolution is doubled, the number of FLOPS required is quadrupled. Therefore, the constraint in (1) indicates that, for any increase in the *ϕ* value, the new number of FLOPS will increase by 2^*ϕ*^. Furthermore, the constant terms must be greater than or equal to one because none of the dimensions should be allowed to be scaled down. The aim of this method [13] is to scale network depth, resolution, and width, such that the accuracy of the network, and the consumption of memory and FLOPS are optimized according to the available resources.

To solidify the concept and prove the effectiveness of the compound scaling method, the authors [13] then developed a mobile-sized baseline network by applying the neural architecture search (a technique used to optimize efficiency and accuracy with respect to FLOPS), which was called the EfficientNet-B0. The model uses inverted residual blocks, consisting of squeeze-and-excitation optimization [37] and swish activation [38]. Swish is defined as

(2) $$\begin{array}{c} \text{Swish}\left(x\right)=x*\text{sigmoid}\left(x\right). \end{array}$$

The inverted residual block was introduced in the MobileNet-v2 architecture [31] and makes use of depth-wise separable convolution to decrease the number of parameters and multiplications needed to execute the network. This modification results in faster computation without adversely affecting performance. The inverted block consists of three major components: a convolutional layer (called the expansion layer) which expands the number of channels to prepare the data for the next layer, a depth-wise convolutional layer, and another convolutional layer (the projection layer) which is meant to project the data from a large number of channels to a small number of channels. The first and last layers of a residual block are connected via a skip connection. Therefore, during fine-tuning, it is imperative to train entire blocks. Disobeying this restriction can damage the way the network learns [39]. The squeeze-and-excition block consists of a global average pooling (GAP), a reshaping, and two convolutional layers. The GAP layer extracts global features, and then the number of channels is squeezed according to a predefined squeeze ratio.

The compound scaling method was then used to create the EfficientNet family which included the versions B1 to B7; the constants *α*,  *β* , and *γ* were fixed; and *ϕ* was scaled.

**Figure 7:**
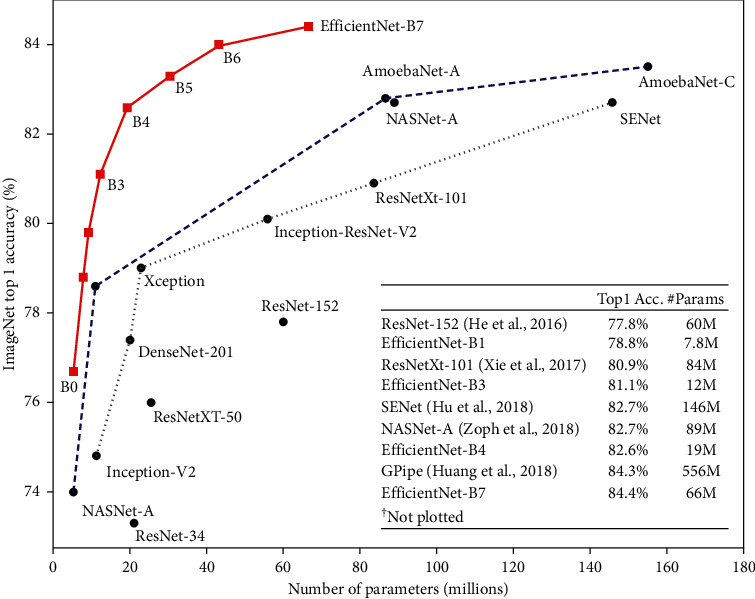
A comparison of EfficientNets with established architectures on the classification of the ImageNet dataset (source: [13]).

The efficacies of the models were tested on the ImageNet dataset and surpassed state-of-the-art convolutional neural networks, with magnitudes being smaller and faster on CPU inference. The outcome (shown in Figure 7) revealed that even though the models have smaller magnitudes than established models in both number of parameters and number of FLOPS, they performed phenomenally.

These models have been successfully used for other histopathology image classification [40–45]. However, at the time of this research, the EfficientNet architecture had not yet been investigated for classification of the ICIAR2018 dataset. We limit our experimentation to the first six EfficientNets due to computational resource restrictions.

### 3.5. Experimental Settings

In order to ensure that results were reproducible, seeds were set for all packages and methods that allowed this. Specifically, a function was used to split the dataset into the training and validation subsets and was seeded; this value was kept constant throughout all experiments. Therefore, all training and validation images across all experiments are the same.

The dataset was divided into train-test sets with split of 85%–15%, as this split produced the highest results and had less difficulty with overfitting. The images in both subsets were stratified, meaning that the classes were equally represented. Stain normalization of the images was accomplished using a package provided by [46].

The implementation of this approach was dependent on the Keras package within the TensorFlow Python library. Specifically, the Keras ImageDataGenerator [47] was used to create augmentation generators for the training and validation data. Publicly available pretrained EfficientNet models were utilized [48].

Each EfficientNet was extended with a global average pooling layer, a dropout layer with a rate of 0.5, followed by a dense layer of 256 neurons with the ReLU activation function, then another dropout layer with a rate of 0.4, and finally a dense layer of 4 neurons with the softmax activation function. The pooling layer performs subsampling. Essentially, the layer reduces the dimensions of the previous layer by combining the neuron clusters into a single neuron [49]. A dropout rate represents the rate at which input units are set to 0 in a dropout layer. If the units are not set to 0, they are updated and scaled up by a value of 1 − 1/dropoutrate to ensure that the sum over all the inputs does not change. Each neuron in a dense layer is connected to every neuron in the previous layer. Therefore, these layers increase the parameter count in a network substantially. The softmax function enables the output of the network to be a vector of probabilities of the input image belonging to each of the four classes. The Adam optimizer was used with a learning rate of 0.0001; this value was empirically tested and found to produce the best results. When the learning rate is too high, the network learns recklessly and is not able to retain information. When the learning rate is too low, the model training progresses very slowly, which means that more epochs, computational resources, and training time are required. Moreover, early stopping with a patience of 10 epochs, reducing learning rate on plateau with a patience of 8 epochs and minimum learning rate set of 0.000001, and model checkpoints were used during each experiment. Therefore, models with the lowest validation losses were saved while training continued until early stopping ended the execution. The batch size was fixed to 16 for the EfficientNets-B0–B4, the size was set to 5 for the B5 and B6 (due to memory constraints), and all models were set to train for 50 epochs. Categorical cross-entropy was employed as the loss function, as it is best suited for multiclass classification. This function is defined as

(3) $$\begin{array}{c} CCE=-\frac{1}{N}\left(\sum_{i=1}^{N}\log\;P_{model}\left[y_{i}\in C_{yi}\right]\right), \end{array}$$

where *N* represents the number of instances and log  *P*_*mo*  *de*  *l*_[*y*_*i*_ ∈ *C*_*yi*_] represents the probability predicted by the model for the *i*^*th*^ instance.

All tests were run on the Google Colaboratory platform which provides 25 GB RAM and a 12 GB NVIDIA Tesla K80 graphics processing unit.

## 4. Results and Discussion

### 4.1. Evaluation Criteria

The performance of each model was evaluated by calculating the precision, recall, F1-score, and accuracy. The equations below represent the manner in which these metrics are determined.

(4) $$\begin{array}{c} \text{Accuracy}=\frac{\text{TP}+\text{TN}}{\text{TP}+\text{TN}+\text{FP}+\text{FN}}\times 100, \end{array}$$

(5) $$\begin{array}{c} \text{Precision}=\frac{\text{TP}}{\text{TP}+\text{FP}}\times 100, \end{array}$$

(6) $$\begin{array}{c} \text{Recall}=\frac{\text{TP}}{\text{TP}+\text{FN}}\times 100, \end{array}$$

(7) $$\begin{array}{c} F1-\text{score}=2\times \frac{\text{Precision}\times \text{Recall}}{\text{Precision}+\text{Recall}}, \end{array}$$

where TP, TN, FP, and FN represent true positives, true negatives, false positives, and false negatives, respectively. The precision and recall scores recorded are averaged over all four classes.

### 4.2. Experimental Results

Table 4 provides the average precision and recall (across the four classes) and the accuracy of each model with the stain normalization methods. Moreover, the average accuracy obtained by the EfficientNet model is calculated.

Accuracy and sensitivity (precision) are of paramount importance in this task. Hence, these metrics will be further analyzed and discussed.

### 4.3. Discussion

#### 4.3.1. Analysis of Results

The Grand Challenge [14] outcomes revealed that the best performing models were pretrained on ImageNet and fine-tuned. Even though many approaches incorporate patch-wise classification to utilize local features, [3, 5, 14] state that using the entire image for classification was found to produce better results. This insight implies that extracting nuclei and tissue organization features is more valuable for deciphering the image classes than nuclei-scale features [14]. The image-level classification in this research observes the ability of the architecture to extract global features in the breast cancer histology images and use it to classify unseen images.

The results of the experiments show that the EfficientNet models perform well on the ICIAR2018 dataset. The Reinhard technique [22] outperforms Macenko in the EfficientNet-B0, -B2, and -B4. The Macenko technique [19] returned the highest accuracy for the EfficientNet-B3, and nonnormalized images performed the best for the EfficientNet-B5 and -B6. For the EfficientNet-B1, both Macenko and Reinhard produced the same results.  Table 5 shows the average accuracy results obtained by each stain normalization method. In this table, the average results of the nonnormalized method are inferior to that of those Reinhard and Macenko techniques. Therefore, it can be concluded that, on average, applying stain normalization to images as part of the preprocessing step is beneficial.

Notably, the EfficientNet-B2 model produced superior results compared to the other six, which indicates that this architecture was the most successful at extracting and learning the global features in the training set. The average accuracy achieved by this model (95.83%) is higher than those reported in the literature. The additional benefit of this approach is that it is simple, requiring fewer parameters, which implies less training time compared to the previous approaches. The use of images normalized with the Reinhard technique returned the highest sensitivity and accuracy for this model, at 98.33%. Following closely is the result from the use of images stained with the Macenko technique, with a sensitivity and accuracy of 96.67%. The EfficientNet-B1 returns identical results for training the images stained with Reinhard and Macenko, with a sensitivity and accuracy of 95.00%. The difference between the number of parameters in the EfficientNets-B1 and -B2, and the input size both models receive are similar. For this specific dataset, the input sizes of 240 × 240 and 260 × 260 are appropriate for successfully extracting global features in the histology images. From Table 4, it is interesting to note that the average accuracy gain seems to increase at first (for EfficientNets-B0–B2), decrease with larger models (for EfficientNets-B3–B5), and then pick up slightly (EfficientNet-B6). The EfficientNet-B0 model has the fewest parameters but does not perform the best. This may be owing to the small input size that the model receives. Resizing the large images to 224×224 may affect the structures in the image, making it more difficult for the model to capture the features. For the larger models that did not perform as well, this may be a consequence of overparameterization relative to the size of the dataset.

Table 6 provides additional insight by showing the models' sensitivity and specificity concerning each class. The *R*, M, and N next to each model name refer to the stain normalization method used on the dataset (Reinhard, Macenko, and nonnormalized). As previously mentioned, high sensitivity is crucial for this problem. It is a widely used metric to determine the proportion of correctly identified positives (as shown in (6)). As expected, the EfficientNet-B2 model shows consistent high sensitivity throughout all four classes; however, it is less sensitive toward the *in situ* carcinoma class. The EfficientNet-B4 and -B5 return the lowest sensitivity to all four classes but especially to the noncarcinoma classes. The sensitivity toward the carcinoma class must be maximized, as an incorrect prediction of this tissue could lead to misdiagnosis and severe consequences. Aresta et al. [14] state that the benign class affects the performance of the networks due to the structural similarity with normal tissue. This class contains more morphological variability than the others, which translates to increased difficulty in learning discriminative features. This statement is consistent with the results reported in this study, as the benign class typically returns the lowest sensitivity relative to the other classes.

#### 4.3.2. Analysis of Accuracy and Loss Curves

**Figure 8:**
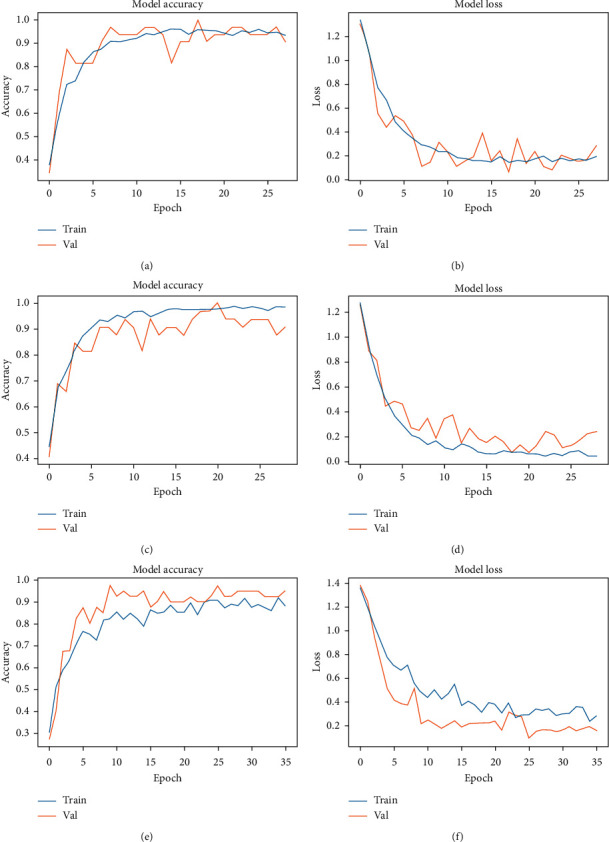
Noteworthy accuracy and loss graphs: (a) EfficientNet-B2 Reinhard accuracy graph; (b) EfficientNet-B2 Reinhard loss graph; (c) EfficientNet-B4 Reinhard accuracy graph; (d) EfficientNet-B4 Reinhard loss graph; (e) EfficientNet-B6 Reinhard accuracy graph; (f) EfficientNet-B6 Reinhard loss graph.

The graphs in Figure 8 show the accuracy and loss curves for three models: the EfficientNet-B2, -B4, and -B6 trained with Reinhard-normalized images. The curves represent the progress of the training and validation accuracy and loss during the training of the models. These metrics are recorded per epoch.

In graphs (a) and (b), both curves are very close to each other. The evident noise in the curves is caused by the data augmentation, as no single image is fed into the convolutional neural network twice. In graphs (c) and (d), it is obvious that the training is proceeding well; however, the validation accuracy and loss are not. This indicates that the B4 architecture is learning features adequately but it is not able to generalize at the same level. Hence, the validation loss curve is extremely noisy and does not fit perfectly. Finally, in graphs (e) and (f), the architecture clearly has difficulty learning the distinguishing features. The training curve in (e) does not increase at the same rate that (a) and (c) do. Similarly, in (f), the validation loss does not descend below 0.2 as it does for the B2 and B4 models in (b) and (d), respectively. A possible reason for the validation loss curve being below the training loss curve in (f) is that the architecture finds the validation set to be unrepresentative of the entire dataset and easier to predict than the training set [52].

**Figure 9:**
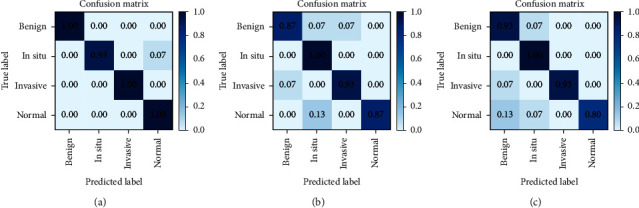
Confusion matrices for the three models: (a) EfficientNet-B2; (b) EfficientNet-B4; (c) EfficientNet-B6.

Figure 9 shows the confusion matrices corresponding to the graphs in Figure 8. From these matrices, it can be seen that the larger models do not predict the noncarcinoma classes well. This indicates that the larger models do not learn the features from the normal and benign classes well.

#### 4.3.3. Comparison with Previous Approaches

The results of this study must be compared with similar approaches to understand the effect of using the EfficientNets instead of other architectures.  Table 7 summarizes the pretrained architectures used in previous approaches and the accuracies obtained for four-class, image-wise classification. It is worth noting that, among the architectures listed in this table, the EfficientNet-B2 has the lowest number of parameters (approximately 8 million) and is computationally cheaper than the other approaches, as it is a lightweight model. The ResNet-50 and Inception-v3 networks also perform well on this dataset, and both architectures have around 23 million parameters. On the other hand, the VGG16 and VGG19 architectures have over 100 million parameters and produce inferior results. This shows that smaller architectures produce higher accuracies for this dataset. The results prove that transfer learning with the EfficientNet-B2 yielded superior results in comparison to other architectures. Both the Reinhard stain normalization technique and Macenko stain normalization technique yielded satisfactory results on the EfficientNet-B2.

#### 4.3.4. Challenges with This Approach

The major challenge faced in these experiments was combatting overfitting. Due to the size of the dataset, overfitting on the large architectures was ensured. Incorporating data augmentation techniques to increase the number of unique samples that the CNN would see was not sufficient, so other forms of regularization had to be explored. These forms include dropout layers, early stopping, and model checkpointing. The appropriate dropout rate was found through a grid search, along with optimal batch size and number of dense layers. Alternative loss and activation functions were investigated but contributed more to overfitting and unstable learning. ReLU was chosen as the activation function for this network as it is computationally efficient, is simple, and has been empirically proven to work well [53].

#### 4.3.5. Limitations of the Study

The main objective of this research was to observe the ability of EfficientNet architecture to classify the images of the ICIAR2018 dataset into four classes: normal, benign, invasive carcinoma, and *in situ* carcinoma. However, classifying the images into the groups *carcinoma* and *noncarcinoma* is valuable and helpful pursuit and should return improved accuracies [5, 17]. This binary classification requires the efficient extraction of local features in the images. The architectural additions proposed in this study perform well in extracting global features for the multiclass classification but do not perform equally in the binary classification. A reasonable explanation for this is that the loss of information caused by resizing images translated to the network having difficulty in locating discriminative local features.

## 5. Conclusion

The EfficientNet family is a set of state-of-the-art convolutional neural networks that achieved preeminent results on established image datasets by carefully balancing the crucial dimensions in a network such that accuracy and efficiency were maximized. The field of medical image analysis suffers from a paucity of publicly available data, which results in researchers having to utilize small and, often, unbalanced datasets. Transfer learning techniques offer support in this area by enabling the reuse of generic features from large source datasets for small target datasets.

In this research, the application of seven versions of EfficientNets with transfer learning for breast cancer histology image classification was investigated. Transfer learning was employed in the form of fine-tuning. The experimental results confirm that this architecture was able to effectively extract and learn the global features in an image, such as the tissue and nuclei organization. These features were then used to classify an image into four classes: normal, benign, invasive carcinoma, and *in situ* carcinoma. Among the seven models tested, the EfficientNet-B2 architecture which has approximately 8 million parameters produced superior results with an accuracy of 98.33% and sensitivity of 98.44%. The results achieved showed that the number of feature maps and the number of parameters (8.0 million) for EfficientNet-B2 are optimum for the research problem in question. Furthermore, the effects of two different stain normalization techniques were observed, and the outcomes were compared to the use of nonnormalized images. From the results of the experiments, no specific stain normalization proved to be superior to the other. Instead, the smaller models performed better with the Reinhard technique, and the larger models performed better with no stain normalization.

For future work, considering the results of this research, it would be interesting to observe the impact of an ensemble of EfficientNets for this application. Furthermore, the possibility of these lightweight architectures performing well on other histology image datasets such as PatchCamelyon would be worth exploring [54]. Since multistage transfer learning has proved to be advantageous [55], investigating this technique for breast cancer histology images may produce similar enhanced accuracies. Therefore, this is also a path worth pursuing. Lastly, we expect to explore whether the proposed model is suitable for incremental learning.

## Figures and Tables

**Table 1:** Summary of related techniques in the literature.

Reference	Dataset	Pretrained	Architecture	Input size	Stain normalization	Image-wise accuracy
Araújo et al. [5]	Bioimaging 2015	No	Custom CNN	512 × 512	Macenko	4-class: 77.8%
						2-class: 80.6%
Vo et al. [17]	Bioimaging 2015	No	3 × Inception-ResNet-v2	600 × 600	Macenko	4-class: 96.4%
				450 × 450		2-class: 99.5%
				300 × 300		
Nawaz et al. [3]	ICIAR2018	Yes	AlexNet	512 × 512	Macenko	81.25%
Ferreria et al. [20]	ICIAR2018	Yes	Inception-ResNet-v2	244 × 244	Nonnormalized	90%
Kassani et al. [21]	ICIAR2018	Yes	VGG16	512 × 512	Macenko	83%
					Reinhard	87%
Kassani et al. [21]	ICIAR2018	Yes	VGG19	512 × 512	Macenko	80%
					Reinhard	84%
Kassani et al. [21]	ICIAR2018	Yes	Inception-ResNet-v2	512 × 512	Macenko	90%
					Reinhard	88%
Kassani et al. [21]	ICIAR2018	Yes	Xception	512 × 512	Macenko	91%
					Reinhard	94%
Kassani et al. [21]	ICIAR2018	Yes	Inception-v3	512 × 512	Macenko	90%
					Reinhard	90%

**Table 2:** Values for data augmentation applied to the stain-normalized images.

Augmentation type	Value
Rescaling	1./255
Rotation range	5°
Width shift range	0.1
Height shift range	0.1
Zoom range	0.3
Horizontal flip	True
Vertical flip	True
Additional rotation	0°, 90°, 180°, 270°

**Table 3:** The number of parameters in each EfficientNet and the recommended input size.

Models	Trainable parameters (million)	Input size
B0	∼4.3	224 × 224
B1	∼6.8	240 × 240
B2	∼8.0	260 × 260
B3	∼11.0	300 × 300
B4	∼17.9	380 × 380
B5	∼28.7	456 × 456
B6	∼41.1	528 × 528

**Table 4:** Results for the EfficientNet architectures with each stain normalization method.

EfficientNet	Stain normalization technique	Precision	Recall	F1-score	Accuracy
B0	Reinhard	93.65	93.33	93.32%	93.33%
	Macenko	91.67	91.67	91.67%	91.67%
	Nonnormalized	92.37	91.67	91.37%	91.67%
					92.9%

B1	Reinhard	94.99	95.00	94.94%	95.00%
	Macenko	94.99	95.00	94.94%	95.00%
	Nonnormalized	93.32	93.33	93.27%	93.33%
					94.7%

B2	Reinhard	**98.44**	**98.33**	**98.33%**	**98.33%**
	Macenko	96.88	96.67	96.66%	96.67%
	Nonnormalized	94.03	93.33	93.29%	93.33%
					**95.3%**

B3	Reinhard	92.50	91.67%	91.62%	91.67%
	Macenko	93.54	93.33	93.27%	93.33%
	Nonnormalized	91.94	91.67	91.66%	91.67%
					92.2%

B4	Reinhard	92.38	91.67	91.69%	91.67%
	Macenko	91.94	91.67	91.66%	91.67%
	Nonnormalized	88.56	88.33	88.31%	88.33%
					90.6%

B5	Reinhard	88.44	88.33	88.14%	88.33%
	Macenko	91.80	91.67	91.53%	91.67%
	Nonnormalized	92.49	91.67	91.56%	91.67%
					90.6%

B6	Reinhard	92.65	91.67	91.67%	91.67%
	Macenko	92.46	91.67	91.68%	91.67%
	Nonnormalized	93.54	93.33	93.39%	93.33%
					92.2%

**Table 5:** The average accuracy results of the stain normalization techniques, Reinhard and Macenko.

Stain normalization technique	Accuracy (avg)
Reinhard	92, 86%
Macenko	93, 10%
Nonnormalized	91, 90%

**Table 6:** Specificity (spec.) and sensitivity (sens.) of the EfficientNet models.

Model	Normal		Benign		In situ		Invasive	
	Spec. (%)	Sens. (%)	Spec. (%)	Sens. (%)	Spec. (%)	Sens. (%)	Spec. (%)	Sens. (%)
B0 (R)	93.33	93.33	87.0	93.33	100	6.7	93.75	100
B1 (R)	92.86	86.7	93.75	100	3.3	93.3	100	100
B2 (R)	93.5	100	100	100	00	93.3	100	100
B3 (M)	100	6.67	86.67	86.7	93.75	100	93.75	100
B4 (R)	100	6.67	92.86	86.7	83.33	100	3.33	93.3
B5 (N)	100	0.00	92.86	86.7	83.33	100	93.75	100
6 (N)	93.33	93.33	87.50	93.33	93.3	93.33	100	93.33

**Table 7:** Comparison with previous approaches using pretrained architectures for classification of the ICIAR2018 dataset.

Reference	Architecture	Stain normalization	Accuracy
Nawaz et al. [3]	AlexNet	Macenko	81.25%
Ferreria et al. [20]	Inception-ResNet-v2	None	90%
Kassani et al. [21]	VGG16	Macenko	83%
		Reinhard	87%
Kassani et al. [21]	VGG19	Macenko	80%
		Reinhard	84%
Kassani et al. [21]	Inception-ResNet-v2	Macenko	90%
		Reinhard	88%
Kassani et al. [21]	Xception	Macenko	91%
		Reinhard	94%
Kassani et al. [21]	Inception-v3	Macenko	90%
		Reinhard	90%
Golatkar et al. [50]	Inception-v3	Vahadane	85%
Vesal et al. [51]	Inception-v3	Reinhard	97.08%
Vesal et al. [51]	ResNet-50	Reinhard	96.66%
Our approach	EfficientNet-B2	Reinhard	**98.33%**
Our approach	EfficientNet-B2	Macenko	96.67%

## Data Availability

The data used to support the findings of this study are included within the article.
